# An AI-Assisted and Self-Powered Smart Robotic Gripper Based on Eco-EGaIn Nanocomposite for Pick-and-Place Operation

**DOI:** 10.3390/nano12081317

**Published:** 2022-04-12

**Authors:** Qi-Lun Goh, Pei-Song Chee, Eng-Hock Lim, Danny Wee-Kiat Ng

**Affiliations:** 1 Department of Mechatronics and Biomedical Engineering, Lee Kong Chian Faculty of Engineering and Science, Universiti Tunku Abdul Rahman, Bandar Sungai Long, Kajang 43000, Selangor, Malaysia; rogergoh2008@1utar.my (Q.-L.G.); ngwk@utar.edu.my (D.W.-K.N.); 2 Department of Electrical and Electronic Engineering, Lee Kong Chian Faculty of Engineering and Science, Universiti Tunku Abdul Rahman, Bandar Sungai Long, Kajang 43000, Selangor, Malaysia

**Keywords:** soft-robotic gripper, self-powered composite sensors, pick and place operation

## Abstract

High compliance and muscle-alike soft robotic grippers have shown promising performance in addressing the challenges in traditional rigid grippers. Nevertheless, a lack of control feedback (gasping speed and contact force) in a grasping operation can result in undetectable slipping and false positioning. In this study, a pneumatically driven and self-powered soft robotic gripper that can recognize the grabbed object is reported. We integrated pressure (P-TENG) and bend (B-TENG) triboelectric sensors into a soft robotic gripper to transduce the features of gripped objects in a pick-and-place operation. Both the P-TENG and B-TENG sensors are fabricated using a porous structure made of soft Ecoflex and Euthethic Gallium-Indium nanocomposite (Eco-EGaIn). The output voltage of this porous setup has been improved by 63%, as compared to the non-porous structure. The developed soft gripper successfully recognizes three different objects, cylinder, cuboid, and pyramid prism, with a good accuracy of 91.67% and has shown its potential to be beneficial in the assembly lines, sorting, VR/AR application, and education training.

## 1. Introduction

With the advancement of industry 4.0, the robotic gripper has become an essential element that can be used for picking and placing objects of any shape, texture, and weight in smart manufacturing. Conventional servomechanism-activated rigid robotic grippers are suffering from poor adaptability. Soft robotic grippers, featuring high compliance with muscle-alike actuation, can address main challenges in traditional rigid grippers [1,2,3,4]. They can form a conformal grasp with fragile or complex geometry objects without damaging them. Numerous actuation principles have been developed to activate the soft grippers, including pneumatic actuation [5,6,7,8], wire-driven actuation [9,10], and actuation based on smart materials, such as shape memory alloys [11,12,13,14], and ionic polymer-metal composite [15]. Among all the above-mentioned actuation principles, the pneumatic actuation outstood other actuation approaches in terms of its flexibility, fast response, and enormous pressure output [16,17]. These advantages have made the pneumatically activated soft gripper a good candidate for the pick-and-place industry. However, most of the reported soft grippers do not have any control feedback (gasping speed and contact force) in their grasping operations. Without the real-time feedback system, it could lead to consequences such as object slippage [18] and incorrect gripping positions that might cause damages to objects.

A camera [19,20,21] has been employed to identify the object feature and location to enable feedback control. Nonetheless, it is challenging when handling objects in a dark environment. In addition, the sensing performance is also constrained by blind spots [22] and a narrow field of view (FOV) [23]. Therefore, the sensors that can be integrated directly into the soft gripper, while detecting continuous gripping motion, have become a major selection. Common strain and pressure sensors, such as elastomer resistive-based and electroactive sensors [24,25], show good compliance with soft structure. However, they pose limitations in sensing performance, i.e., the power source requirement and back relaxation phenomenon. Therefore, soft sensors are required to be developed with self-powering capability and low maintenance. A triboelectric sensor that works based on electrostatic induction between two materials can be an alternative for soft gripper integration [26,27]. The sensor produces an output voltage in response to the structural deformation and separation distance of materials [28], enabling a more straightforward approach in designing its signal processing circuit. Moreover, the triboelectric layer can be constructed by using deformable materials such as Polydimethylsiloxane (PDMS) [29] and hydrogel [30]. A TENG-based sensor’s output power is highly reliant on the contact separation distance between two triboelectric layers. Recently, surface modification on the TENG structure has been extensively explored for better sensitivity in a small contact separation distance. S. Chen et al. proposed integrating a micropyramid structure on the surface of the gripper to increase its contact area [31]. Although the microstructure improves the TENG output, it wears out easily, which is caused by repetitive contact frictions between the triboelectric layers [32]. Another approach to increase the surface area is to integrate micropores [33,34] into the triboelectric layer. This can be achieved by fabricating a sponge-based structure within the triboelectric layer, which helps to trap triboelectric charges. However, there is limited research on applying the micro-structured triboelectric material for soft robotic applications.

In this work, we report a pneumatically driven and self-powered soft robotic gripper that is capable of sensing a grabbed object based on the triboelectric principle. The proposed soft gripper is integrated with the pressure (P-TENG) and bend (B-TENG) triboelectric sensors, which are fabricated using the soft Ecoflex and Euthethic Gallium-Indium nanocomposite (Eco-EGaIn). This nanocomposite enables the triboelectric sensors to comply with the soft gripper. To improve their sensitivities, micropores structures are formed in the Eco-EGaIn composite layer. The detected output voltages from the sensors are then fed into an artificial intelligence (AI) algorithm for object recognition. The developed soft gripper has successfully recognized three different objects, cylinder, cuboid, and pyramid prism, in a pick-and-place operation. The proposed smart gripper can benefit assembly lines, sorting, VR/AR application, and education training.

## 2. Materials and Methods

### 2.1. Design of the Soft Robotic Gripper

**Figure 1:**
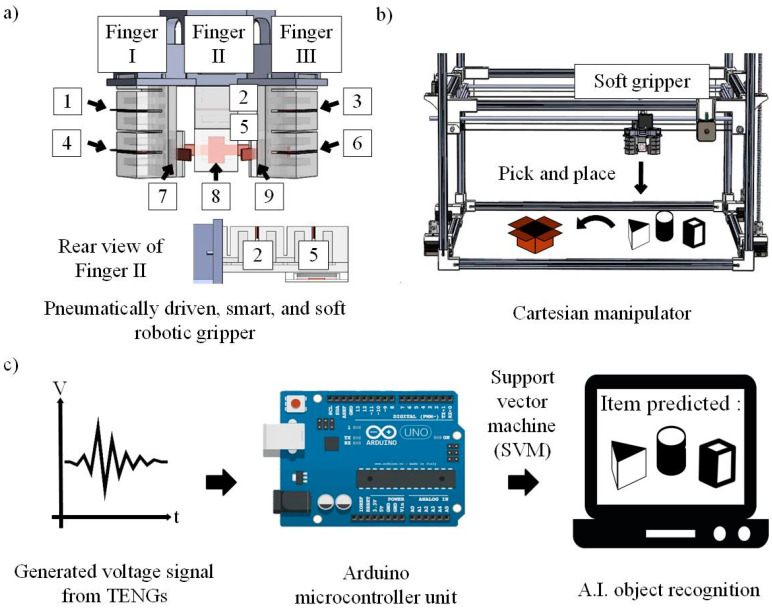
(**a**) Schematic illustration of the soft robotic gripper integrated with inner TENGs strips (components 1–6) for measuring the bending angle and the soft TENGs (components 7–9) at each fingertip to detect the contact force. (**b**) The soft gripper was attached to a 3-axis cartesian manipulator to perform pick-and-place operations. (**c**) Process flow for data acquisition to perform object classification.

The soft robotic gripper (in Figure 1a) is designed by integrating three soft elastomer fingers (Finger I, II, and III), a pneumatic-control valve, and an inner TENGs strips (components 1–6 in Figure 1a) for measuring the bending angle, and the soft TENGs (components 7–9 in Figure 1a) at each fingertip to detect the contact force. Each soft elastomer finger is made of Ecoflex 00–50 (Smooth-On, Easton, PA, USA). Its outer surface is designed with corrugated structures to enable bending movements based on the expansion of the embedded pneumatic chamber at an applied pressure. This design provides a stable grasping operation as it does not require precision routing for actuation, as compared to a wire-driven mechanism [35,36,37]. The three soft elastomer fingers were assembled to a 3D printed holder and actuated using a pneumatic system (Eurox EAX-5010, Selangor, Malaysia). The full soft robotic gripper was then attached to a 3-axis cartesian manipulator (Figure 1b) to perform the pick-and-place operation, which is controlled by using bipolar stepper motors (NEMA 17 HS4401, Changzhou, China). The movement of the cartesian manipulator was programmed using Printrun (version 20140406, Kliment Yanev) an open-source software. Upon grasping the object, the electrical output from the sensors will be collected by a microcontroller unit (Arduino Mega 2560, SparkFun Electronics, Niwot, CO, USA) and sent to a computer-based system to perform object classification based on a support vector machine (SVM) learning algorithm (Figure 1c).

### 2.2. Fabrication of Sensors

**Figure 2:**
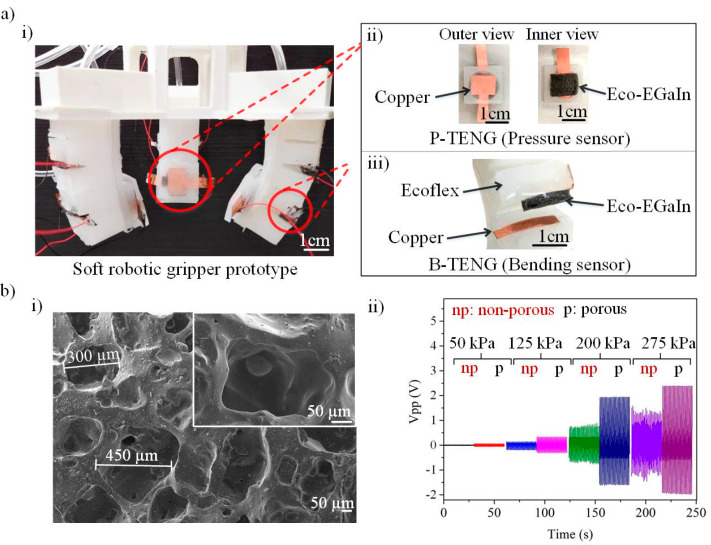
(**a**) (**i**) Photograph of the soft robotic gripper prototype. (**ii**) Schematic illustration for the pressure sensor (P-TENG) and (**iii**) bend sensor (B-TENG). (**b**) (**i**) Scanning electron microscope (SEM) image of the Eco-EGaIn nanocomposite. (**ii**) Output voltage comparison between the porous and non-porous Eco-EGaIn triboelectric layers at 1 Hz compression pressure.

Figure 2a(i) depicts the fabricated soft gripper that is made using the soft lithography process. The placements of the P-TENG and B-TENG sensors in the soft gripper are illustrated in Figure 2a(i,ii) respectively. In the Supplementary Materials, Figure S1 shows the assembly process of the P-TENG and B-TENG components. The employment of a porous structure is a proven approach for improving the contact surface’s area [38,39]. Improvement in the contact surface area induces a higher level of surface charge transfer in the triboelectric layer during the contact–separation operation, which has, in turn, increased its sensitivity. This is also supported by the literature [40], showing that the compression of the micropores induces extra charges due to electrostatic effects. Inspired by this finding, we fabricate our TENG sensors by using sponge architecture. This can be achieved by mixing 3.9 g of sodium chloride (NaCl) particles (Merck & Co., Inc., New Jersey, NJ, USA) in a 1.3 g Ecoflex 00–50 solution. The mixture was mixed with 0.8 g of EGaIn (68.5% Ga, 21.5% In, 10% Sn) to form a conductive elastomer, Eco-EGaIn. The mixture, Eco-EGaIn, was cast in a mold and cured at 70 °C for 6 h. Later, Eco-EGaIn was immersed in deionized water (DI) at 60 °C with continuous stirring for 8 h to dissolve the pre-mixed NaCl particles. The NaCl particles act as sacrificial templates for the porous formation in the composite Eco-EGaIn material. Figure 2b(i) shows the SEM image of the cross-sectional porous Eco-EGaIn layer. The average diameters of the porous structures were measured using a Java-based image processing program, ImageJ (version 1.53n, University of Wisconsin, Madison, WI, USA) and were found to be approximately 330 µm. To investigate the effects of porosity on the sensitivity of the composite material, different porosities were fabricated by adjusting the concentration of NaCl in the Eco-EGaIn nanocomposite. The SEM images for different NaCl concentrations (0%, 25%, 45%, and 65%) are presented in Supplementary Materials, Figure S2. It is notable that porosity increases with NaCl concentrations. Figure 2b(ii) compares the TENG outputs for the porous and non-porous structures at an applied pressure of 50 kPa, 125 kPa, 200 kPa, and 275 kPa with 1 Hz compression frequency. It can be observed that the sponge-structured TENG patches show improvements for all pressure ranges, of which 275 kPa shows the greatest improvement of 63%. This improved output voltage generated from the TENG patches shows better sensitivity, which helps to increase the pressure sensing range of the pick-and-place operation. On top of that, it provides a high signal-to-noise ratio measurement, eliminating the involvement of a complex signal filtering circuitry. 

## 3. Results and Discussion

### 3.1. Single-Electrode Mode P-TENG Sensor

**Figure 3:**
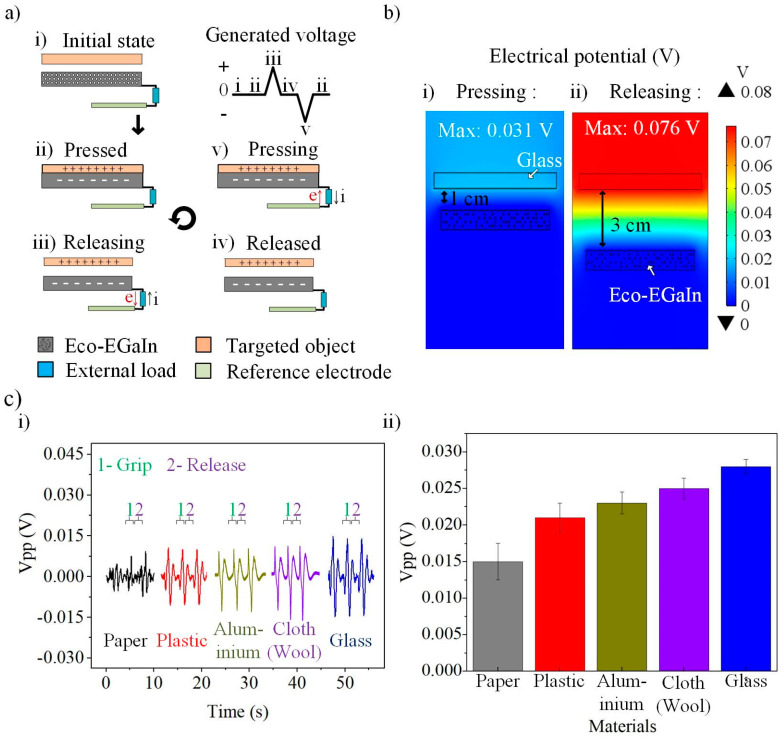
(**a**) Working mechanism of the P-TENG in a single electrode mode, (**i**) initial state, (**ii**) pressed, (**iii**) releasing, (**iv**) released, and (**v**) pressing. (**b**) Simulation of the potential distribution of P-TENG when (**i**) pressing and (**ii**) releasing. (**c**) (**i**) Dynamic characteristic and (**ii**) static characteristic of the P-TENG for five different materials.

As schematically illustrated in Figure 3a, P-TENG is configured in a single-electrode mode by connecting Eco-EGaIn to a reference electrode. At the initial state (Figure 3a(i)), no charge transfer is induced. When the soft gripper grasps an object (Figure 3a(ii)), the separation distance between P-TENG and the object reduces, which results in Triboelectric charges transfering over the two contacted surfaces. The negative charges accumulate on the P-TENG triboelectric layer (Eco-EGaIn), while the positive charges are on the grasped object. A potential gradient is formed between the conductive Eco-EGaIn and the reference electrodes in the release process. The free electrons are drawn from Eco-EGaIn to the reference electrode through an external load (Figure 3a(iii)), forming a positive output peak until equilibrium is reached when the two surfaces are completely separated apart (Figure 3a(iv)). In the next contact between the targeted object and the P-TENG triboelectric layer, it breaks the equilibrium state and induces electrons to flow in the opposite direction, resulting in a negative peak, as shown in Figure 3a(v).

The contact-separation charge transfer mechanism is further examined using COMSOL Multiphysics, a finite element modelling tool (Figure 3b). A glass material was set as the positive triboelectric layer with its charged density of 70 pC/m^2^. It can be found that the maximum electrical potential differences of the glass and the P-TENG increase from 0.031 V to 0.076 V when their separation distance is enlarged from 1 cm to 3 cm during the pressing (Figure 3b(i)) and releasing ((Figure 3b(ii)) states. An experiment was conducted by gripping five different materials (paper, plastic, aluminium, cloth (wool), and glass), and their corresponding signal waveforms are recorded in Figure 3c(i). All materials have the same shape and dimension. As observed in Figure 3c(ii), the five materials show different peak voltages, V_pp_, with the glass material showing the highest voltage output. This output trend is attributed to the high potential difference of the glass and Eco-EGaIn (the build-up material of the P-TENG) in the Triboelectric series [41]. 

### 3.2. Double-Electrodes Mode P-TENG Sensor

**Figure 4:**
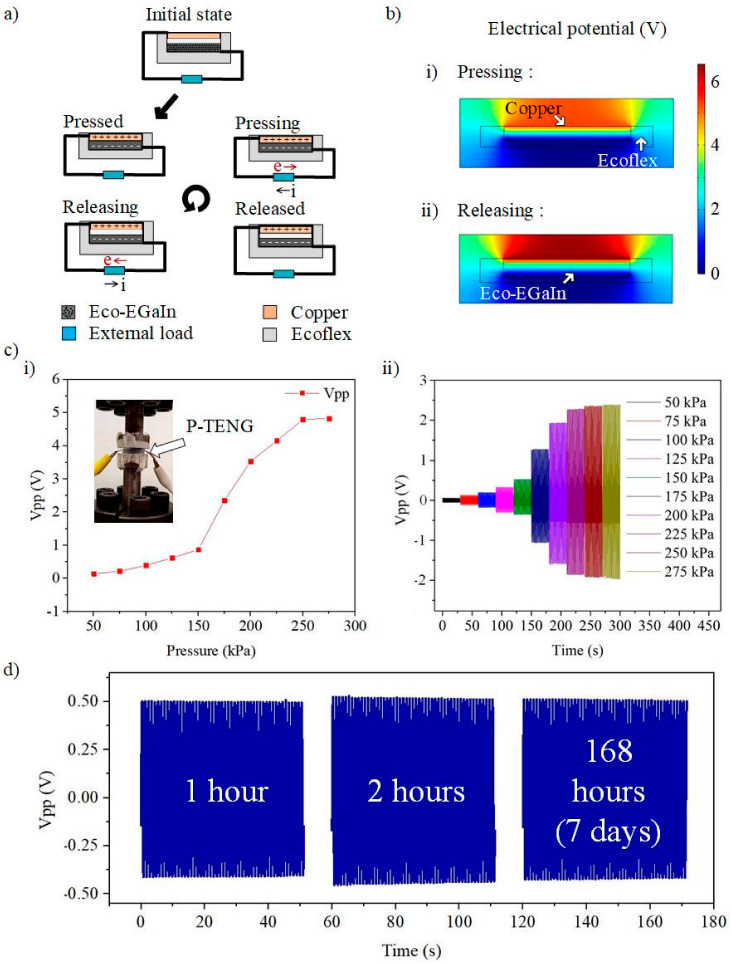
(**a**) Working mechanism of the P-TENG in dual electrodes mode. (**b**) Simulation of the potential distribution in the P-TENG when (**i**) releasing and (**ii**) pressing. (**c**) The output voltage of the P-TENG in (**i**) steady-state and (**ii**) transient. (**d**) Robustness test of P-TENG.

We design double-electrodes P-TENG by sandwiching an Ecoflex between an Eco-EGaIn nanocomposite and a flexible copper. The Ecoflex functions as the separation gap for inducing triboelectrification. The charge transfer mechanism in the triboelectric material is almost similar to that of the single-electrode mode, except in this mode, another end of the load is connected to the flexible copper to serve as the positive triboelectric layer (Figure 4a). Compared with the single-electrode mode, for which its output voltage is highly dependent on the sensing material, this double-electrode mode enables the contact pressure of the gripped object to be quantified based on the output voltage of P-TENG in the pick-and-place operation. We further examine the potential distribution on P-TENG using COMSOL Multiphysics, as shown in Figure 4b(i,ii). The potential contours significantly indicate that the potential difference has been induced when the flexible copper is separated from the Eco-EGaIn nanocomposite, driving the flow of electrons through the external circuit.

We conducted a pressure dependency test of P-TENG using the Shimadzu Servo Pulser E-series (Shimadzu, Kyoto, Japan), with the experimental setup shown in the inset of Figure 4c(i). The output voltage from the P-TENG was acquired using an NI Elvis board (NI-ELVIS Series II, Austin, TX, USA). Figure 4c(i) summarizes the correlation between the input pressure and the output open-circuit voltage. A maximum open-circuit output voltage of 4.8 V_pp_ was observed at 250 kPa. The output voltage saturates when the input pressure exceeds 250 kPa as the maximum separation distance is reached. Figure 4c(ii) shows the transient response of P-TENG. The highly repetitive pattern proves the consistency and reliability of the measurement. It is also worth noting that symmetrical positive and negative peaks indicate the high elasticity of Ecoflex (material used to build the separation distances) without any hysteresis. We further examine the robustness and stability of P-TENG by measuring its output voltage for 1 h, 2 h, and 168 h (7 days) under an operating frequency and compression force of 1 Hz and 150 kPa, respectively. The response of P-TENG is presented in Figure 4d. It shows no significant deterioration in the output voltage after long operating hours, ensuring its good reliability and durability.

### 3.3. Characterisation of B-TENG Sensor

**Figure 5:**
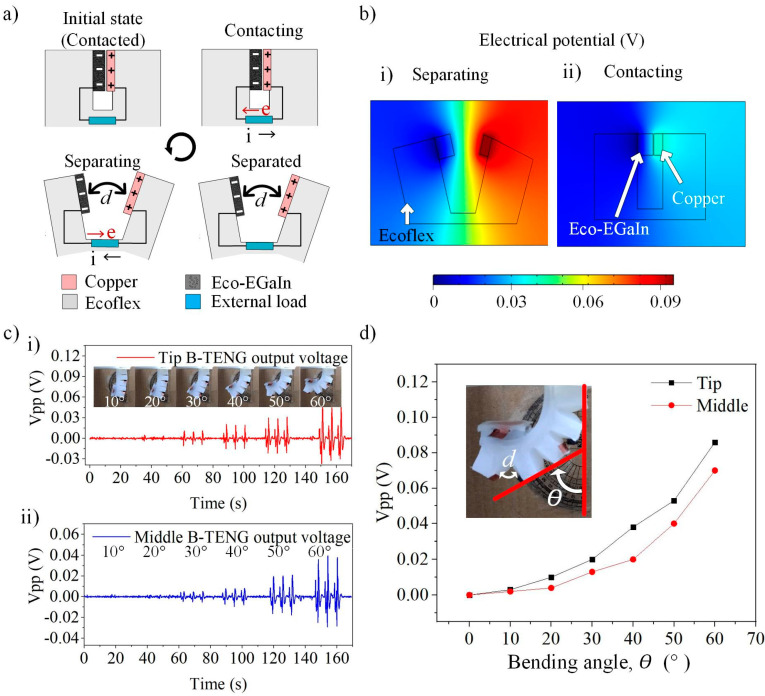
(**a**) The operation mechanism of B-TENG. (**b**) Simulation of the potential distribution in B-TENG when the triboelectric strips are separating (**i**) and contacting (**ii**). (**c**) The triboelectric output is generated at the (**i**) tip of B-TENG under a bending angle of 10° to 60°. The inset shows finger bending profile (**ii**) at the middle of B-TENG. (**d**) The output voltage of B-TENGs at a different bending angles.

The B-TENG sensor is designed with two triboelectric strips (Eco-EGaIn and copper) placed on the corrugated structures of the finger. The Eco-EGaIn strip is bonded on one end of the chamber surface, while the other flexible copper electrode strip is bonded on its opposite. Figure 5a describes the working mechanism of B-TENG integrated onto the corrugated structures of the soft finger. The separation distance between the triboelectric strips is defined as d. Both the triboelectric strips separate when the soft finger bends under pneumatic actuation. The separation distance between the strips, d, increases with further bending of the finger, making the Eco-EGaIn strip negatively charged and the flexible copper trip positively charged due to their affinity in attracting and losing electrons. The simulation result in Figure 5b shows the charge distribution when the two triboelectric strips are separating (Figure 5b(i)) and contacting (Figure 5b(ii)). In a grasping operation, the contact position of the object and the finger varies with their size. Figure 5c shows the temporal response of the finger for three repetitive bends and unbending cycles. It can be observed that the three repetitive output voltage produce a symmetrical and stable output. The output voltage increases with the bending angle of the soft finger, which validates that B-TENG has been successfully working as a sensor for detecting the bending degree. Meanwhile, the performance of the B-TENG sensors by placing them at the tip and middle of the fingers are shown in Figure 5c(i,ii), respectively. The bending profile of the soft finger is shown in the inset of Figure 5c(i), with the bending angles, *θ*, from 10° to 60°. Figure 5d shows that the B-TENG sensors at the tip have a higher output than those at the middle, indicating a larger separation distance between the triboelectric strips, d, at the same bending angle, *θ*, from the soft finger. This behavior explores the potential of B-TENG in sensing the contact position of the grasped object.

### 3.4. TENG Sensing and Object Recognition

**Figure 6:**
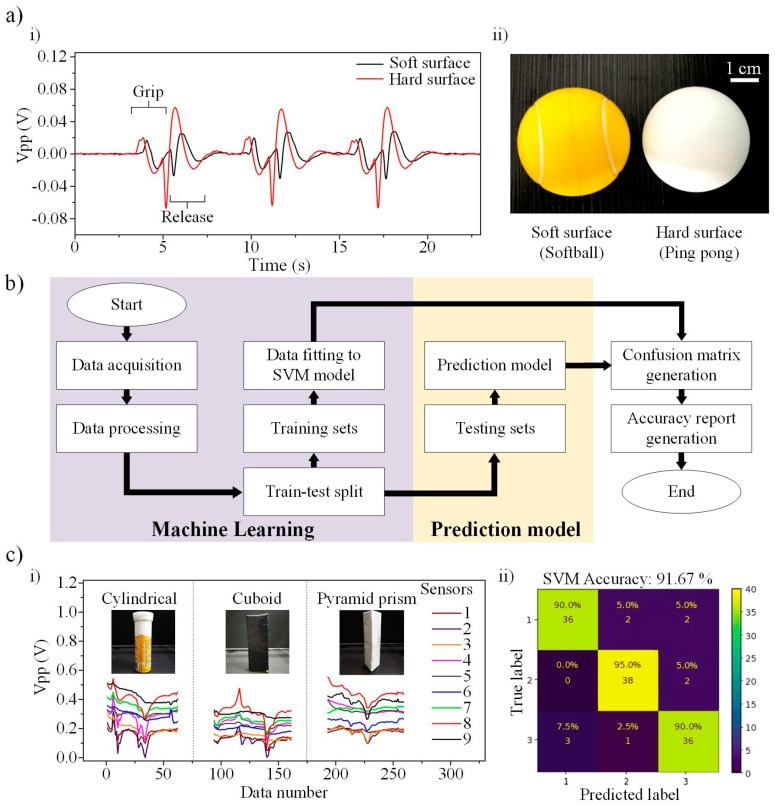
(**a**) (**i**) The output voltage of the P-TENG when gripping two sphere-shaped objects (diameter = 4 mm) with different surface hardness. (**ii**) Inset photograph of the two sphere-shaped objects with different surface hardness. (**b**) Flowchart of the support vector machine learning algorithm. (**c**) (**i**) The voltage output of the sensors when gripping different objects. (**c**) (**ii**) The confusion map of the object recognition results.

The sensing functionality of P-TENG under pneumatic actuation is first tested by grasping and releasing a soft and hard object. Figure 6a(i) shows the output signals when gripping and releasing the two objects. Figure 6a(ii) illustrates the photograph of the two objects. A 4 cm diameter softball (softer surface) and ping pong ball (harder surface) were used in the experiments. The output signal shows a similar pattern for both objects, with the exceptiong that the hardball material exhibits a higher magnitude than the softer material. This is due to the high compression pressure exerted on P-TENG when gripping the hard object, resulting in a more significant change in the separation distance between the two triboelectric layers. The viscoelastic effect of the soft surface acts as a damper to suppress the compression pressure. It is also worth noting that the positive peak in the release state is higher than the grasp state. This phenomenon can be related to the slow air injection rate by the regulator in the gripping action. The air is vacuumed out fast and triggers a larger positive peak. In addition to the static characterization, we further examine the soft gripper for its dynamic behaviour by picking and placing an egg in the Supplementary Materials, Video S1. 

Machine learning (ML) is an advanced technology that can be employed for gripped object recognition by processing massive input signals and extracting features from a data set based on an algorithm. Amongst the algorithms (such as principal component analysis, random forest, etc.), the support vector machine (SVM) is an efficient supervised learning model that can be used for classification, and it has been widely applied for analyzing triboelectric output signals with high accuracy. In this project, a customized SVM-based recognition platform has been developed and applied for pick-and-place operations in the soft gripper. The experimental setup of the soft gripper on the 3D Cartesian axis is mentioned in Section 2.1. Figure 6b shows the flow chart of the SVM operation for object recognition. First, the output voltage signals from the P-TENG and B-TENG sensors for different shapes, including a cylinder, a cuboid, and a pyramid prism, are collected using a microcontroller unit (Arduino Mega 2560) and analyzed accordingly by repeating picking and placing motions for 200 times each. The collected raw voltage data for the three objects in the time domain are used as the features for SVM training. There are 9 (number of sensors) × 70 (data length for each sensor) = 630 features for each object during the grasping motion. The feature includes information of the contact pressure, speed, contact duration, etc. The data were then split into training and testing data sets with a ratio of 8:2, consisting of 160 training data sets and 40 testing data sets for each object. The SVM model learns the features from the training data sets and uses the testing data sets to examine the prediction accuracy of the grasped objects. Finally, the accuracy of the prediction results is shown in a confusion matrix. The signals collected from the sensors for the three objects are visualized in Figure 6c(i). The confusion matrix in Figure 6c(ii) shows a high accuracy of 91.76%, which is higher than several reported studies with only approximately 84–90% accuracy [42,43,44,45]. Video S2 in the Supplementary Materials info demonstrates that the soft gripper has successfully recognized the objects in the pick-and-place operation. The result shows that the developed soft gripper can identify the gripped objects accurately, and it can be further applied to an assembly line for warehouse management in a next-generation smart factory.

## 4. Conclusions

An AI-driven soft robotic gripper that is integrated with the self-powered P-TENG and B-TENG sensors has been demonstrated for pick-and-place operations. The output of the sensors can be improved as much as 63% by fabricating the triboelectric layer in a sponge structure using the Eco-EGaIn nanocomposite. The P-TENG sensor can detect an input pressure ranging from 50 kPa to 275 kPa. It shows high robustness in detecting 150 kPa compression force for 7 days without noticeable deterioration. B-TENG, on the other hand, offers a scalable measurement of the bending angle, with a maximum angle change of 60 °. By feeding real-time data from both the P-TENG and B-TENG sensors into an SVM learning algorithm, the developed soft gripper has successfully recognized various objects with 91.67% accuracy. Future work will further expand the capability of the soft gripper in virtual reality applications.

## Data Availability

Not applicable.

## Supplementary Materials

The following supporting information can be downloaded at: https://www.mdpi.com/article/10.3390/nano12081317/s1, Figure S1. Schematic illustration shows the assembly of (i) P-TENG (Pressure sensor) and (ii) B-TENG (Bending sensor). Figure S2: Cross-sectional SEM images of the conductive Eco-EGaIn with (i) 0% concentration of NaCl particles, (ii) 25% concentration of NaCl particles, (iii) 45% concentration of NaCl particles, and (iv) 65% concentration of NaCl particles. Code S1: Data acquisition code for object recognition application (Arduino IDE), Video S1: Demonstration of pick-and-place operations. Video S2: A.I. object recognition in a pick-and-place operation.
