# Supplementary material for: An AI-Assisted and Self-Powered Smart Robotic Gripper Based on Eco-EGaIn Nanocomposite for Pick-and-Place Operation
Source: Nanomaterials (Basel). 2022 Apr 12;12(8):1317. doi: 10.3390/nano12081317 (PMC9030518; doi:10.3390/nano12081317)
Supplement: Supplementary file 1 [file nanomaterials-12-01317-s001.zip › nanomaterials-1648727-supplementary/nanomaterials-1648727 SI/Supporting Materials.pdf]

Supporting Documents

An AI-Assisted and Self-Powered Smart Robotic Gripper Based on Eco-EGaIn Nanocomposite for Pick-and-Place Operation

Qi-Lun Goh [1], Pei-Song Chee [1,*], Eng-Hock Lim [2,*] and Danny Wee-Kiat Ng [1]

[1] Department of Mechatronics and Biomedical Engineering, Lee Kong Chian Faculty of Engineering and Science, Universiti Tunku Abdul Rahman, Bandar Sungai Long, 43000, Kajang, Selangor, Malaysia; rogergoh2008@1utar.my (Q.L.G.); ngwk@utar.edu.my (D.W.K.N.)

[2] Department of Electrical and Electronic Engineering, Lee Kong Chian Faculty of Engineering and Science, Universiti Tunku Abdul Rahman, Bandar Sungai Long, 43000, Kajang, Selangor, Malaysia

* Correspondence: cheeps@utar.edu.my (P.-S.C.); limeh@utar.edu.my (E.H.L.)

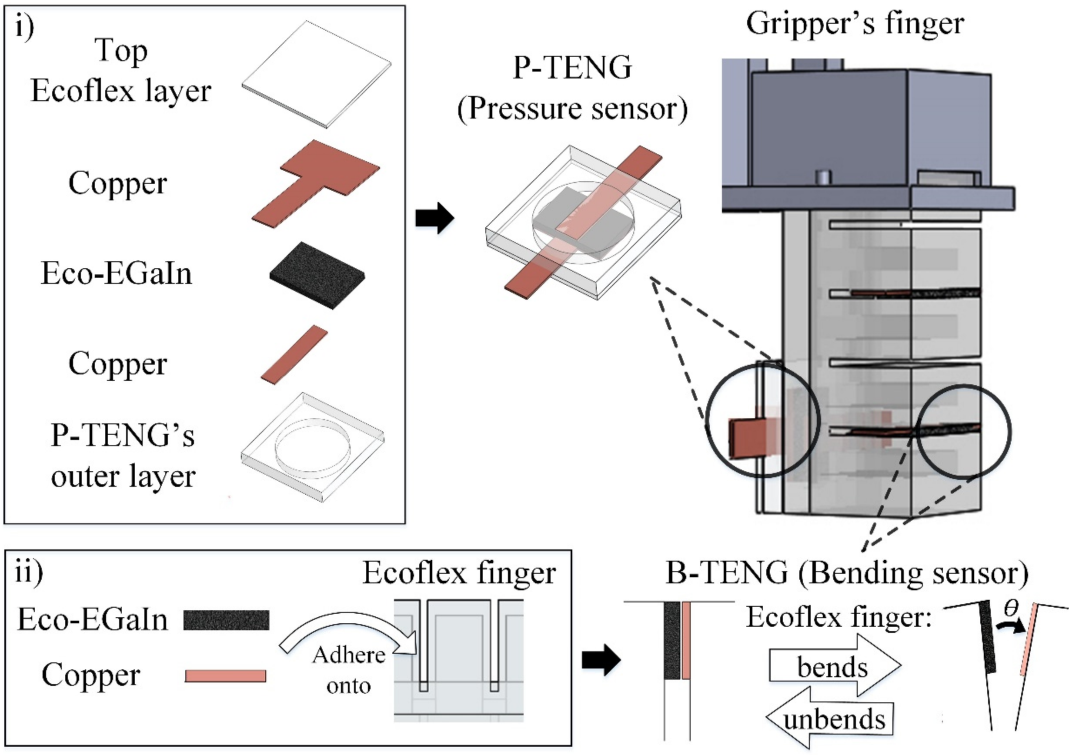

Figure S1. Schematic illustration shows the assembly of the (**i**) P-TENG (Pressure), (**ii**) B-TENG (Bending) sensors.

Figure S1 illustrates the assembly diagram for both P-TENG (Figure S1(i)) and B-TENG (Figure S1(ii)) sensors. The P-TENG sensor is constructed by bonding two triboelectric layers: porous-structured Eco-EGaIn, and flexible copper-clad polyimide between two Ecoflex layers, which serve as the top and outer layer. The B-TENG sensor, on the other hand, is fabricated by inserting a porous Eco-EGaIn layer and the copper-clad polyimide in a cavity of the finger. The bend and unbend movement of the soft finger at a pneumatic actuation change their separation distance.

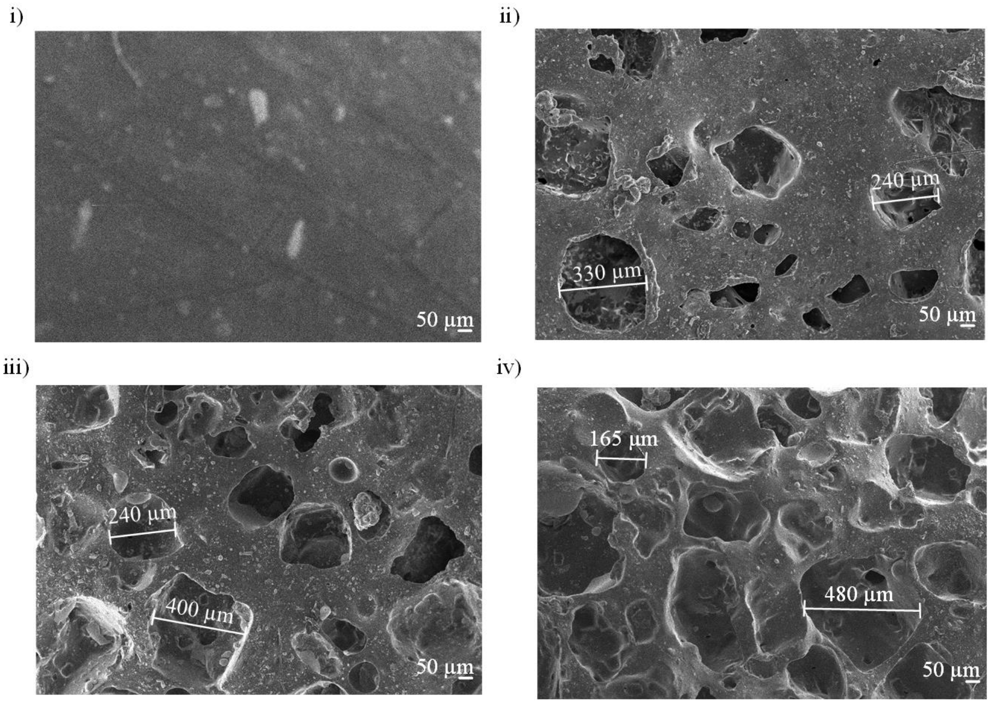

Figure S2. Cross-sectional SEM images of the conductive Eco-EGaIn with (**i**) 0% concentration of NaCl particles, (**ii**) 25% concentration of NaCl particles, (**iii**) 45% concentration of NaCl particles, and (**iv**) 65% concentration of NaCl particles.

Figure S2 shows the cross-sectional SEM images of the conductive Eco-EGaIn with four different concentrations: 0% (Figure S2(i)), 25% (Figure S2(ii)), 45% (Figure S2(iii)) and 65% (Figure S2(iv)). As can be observed in the figures, the porosity in the Eco-EGaIn increases with the NaCl concentration in a fixed area.

Code S1. Data acquisition codes for object recognition application (Arduino IDE).

```
#include <Arduino.h>

#define TIMER_INTERRUPT_DEBUG 0
#define _TIMERINTERRUPT_LOGLEVEL_ 0

#define USE_TIMER_1 false
#define USE_TIMER_2 false
#define USE_TIMER_3 false
#define USE_TIMER_4 false
#define USE_TIMER_5 true

#define TIMER5_INTERVAL_MS 3L

#include "TimerInterrupt.h"

#define ADC_VCC_REF 0x40
#define MUX_GND 0x1F
#define LED_PIN 13

int LED = 13;
int button = 2;
int buttonState = LOW;
int prevState = LOW;
unsigned long lastDebounceTime = 0;
unsigned long debounceDelay = 60;
int incomingByte = 0;


typedef void (*conversion_ready_callback)(unsigned int *);

class Conversion
{
public:
  Conversion()
  {
  }
  void init(conversion_ready_callback callback)
  {
    // ADC Setup
    ADCSRA = 0x0F;
    ADCSRB = 0x00;
    ADMUX = ADC_VCC_REF;
    DIDR0 = 0xFF;
    DIDR1 = 0xFF;
    ADCSRA |= 0x80;
    this->callback = callback;
  }
  void trigger()
  {
    if (enabled == false)
      return;

    if ((ADCSRA & 0x40) != 0x40)
    {
      ADMUX &= 0xE0;
      ADMUX |= 0x1F;
      ADCSRB &= 0xF7;
      ADCSRA |= 0x40;
      discharging = true;
    }
  }
```

```
  void run()
  {
    if (enabled == false)
      return;
    if (discharging)
    {
      ADMUX &= 0xE0;
      ADMUX |= channel < 8 ? channel : channel - 8;
      if (channel < 8)
        ADCSRB &= 0xF7;
      else
        ADCSRB |= 0x08;
      ADCSRA |= 0x40;
      discharging = false;
    }
    else
    {
      ADMUX &= 0xE0;
      ADMUX |= 0x1F;
      ADCSRB &= 0xF7;
      discharging = true;
      *(buffer++) = ADC;
      if (++channel == 13)
      {
        channel = 0;
        if (bufferInUsed == 0)
        {
          callback(buffer0);
          bufferInUsed = 1;
          buffer = buffer1;
        }
        else
        {
          callback(buffer1);
          bufferInUsed = 0;
          buffer = buffer0;
        }
      }
    }
  }

  void start()
  {
    channel = 0;
    discharging = false;
    bufferInUsed = 0;
    buffer = buffer0;
    enabled = true;
  }

  void stop()
  {
    enabled = false;
  }

private:
  bool enabled = false;
  bool discharging = false;
  unsigned int bufferInUsed = 0;
  unsigned int channel = 0;
  unsigned int buffer0[16];
  unsigned int buffer1[16];
  unsigned int *buffer = buffer0;
```

```
  conversion_ready_callback callback;
};

Conversion conversion;
unsigned int adcData[16];
bool conversionCycleComplete = false;

ISR(ADC_vect)
{
  conversion.run();
}

void timerHandler()
{
  static bool triggered = false;
  static int count = 0;
  static int halfSecond = 500 / TIMER5_INTERVAL_MS;
  if (halfSecond == ++count)
  {
    digitalWrite(LED_PIN, triggered ? HIGH : LOW);
    triggered = !triggered;
    count = 0;
  }
  conversion.trigger();
}

void conversionReady(unsigned int *data)
{
  conversionCycleComplete = true;
  memcpy(adcData, data, 32);
}

void setup()
{
  MCUCR = (1 << JTD);
  MCUCR = (1 << JTD);
  Serial.begin(230400);
  pinMode(LED_PIN, OUTPUT);
  while (!Serial)
  {
  }
  digitalWrite(LED_PIN, LOW);
  ITimer5.init();
  ITimer5.attachInterruptInterval(TIMER5_INTERVAL_MS, timerHandler);
  conversion.init(conversionReady);
}

void loop()
{
   buttonState = digitalRead(button);

  if ((millis() - lastDebounceTime) > debounceDelay){
    if(buttonState == HIGH && prevState == LOW){
      lastDebounceTime = millis();
      prevState = HIGH;
      Serial.println("1"); //send start sentinel to PC
    }
    else if(buttonState == LOW){
      prevState = LOW;
      lastDebounceTime = millis();
    }

  {
    char value = Serial.read();
```

```
    if (value == '1')
    {
      conversion.start();
    }
    else if (value == 'S')
    {
      conversion.stop();
    }
  }
  if (conversionCycleComplete)
  {
    for (int i = 0; i < 13; i++)
    {
      //Serial.print(adcData[i]);
      Serial.print("S= ");
      Serial.print(adcData[i]);
      Serial.print(" ");
    }
    Serial.println(adcData[13]);
    conversionCycleComplete = false;
  }


  }
}
```
